# A Study on Selected Physiological Parameters of Plants Grown Under Lithium Supplementation

**DOI:** 10.1007/s12011-012-9435-4

**Published:** 2012-05-11

**Authors:** Barbara Hawrylak-Nowak, Monika Kalinowska, Maria Szymańska

**Affiliations:** Department of Plant Physiology, Faculty of Horticulture and Landscape Architecture, Lublin University of Life Sciences, Akademicka 15, 20-950 Lublin, Poland

**Keywords:** Assimilation pigments, Lipid peroxidation, Lithium, Maize, Sunflower, Toxicity symptoms

## Abstract

Exposure of sunflower and maize plants to increasing concentrations of lithium (0–50 mg Li dm^−3^) in a nutrient solution induced changes in biomass, leaf area and photosynthetic pigment accumulation, as well as levels of lipid peroxidation. The highest applied lithium dose (50 mg Li dm^−3^) evoked a significant reduction in the shoot biomass for both examined species, as well as necrotic spots and a reduction of the leaf area in sunflower plants. An enrichment of a nutrient solution with 5–50 mg Li dm^−3^ did not significantly affect chlorophylls *a* and *b* and the carotenoid content in sunflower plants. However, in maize, a significant decrease in all pigment content under highest used lithium concentration was noted. The levels of lipid peroxidation of the cell membranes in leaves of sunflower plants and the roots of maize increased significantly in the presence of 50 mg Li dm^−3^, which suggests disturbances of the membrane integrity and pro-oxidant properties of the excess lithium ions. Nonetheless, in maize, an increase of shoot biomass and leaf area in the presence of 5 mg Li dm^−3^ was found. An analysis of the metal content indicated that lithium accumulated significantly in sunflower and maize shoots in a dose-dependent manner, but differences occurred between species. The sunflower plants accumulated considerably greater amounts of this metal than maize. The potassium content in shoots remained unchanged under lithium treatments, except for a significant increase in the potassium levels for sunflower plants grown in the presence of 50 mg Li dm^−3^. These results suggest that lithium at 50 mg Li dm^−3^ is toxic to both plant species, but the symptoms of toxicity are species-specific. Moreover, the lithium influence on plants is dose-dependent and its ions can exert toxicity at high concentrations (50 mg Li dm^−3^) or stimulate growth at low concentrations (5 mg Li dm^−3^).

## Introduction

Lithium (Li), which is the lightest of the alkali metals, is present in the earth’s crust to an extent of about 0.006 wt%. It is the 27th most abundant element in nature [1]. Lithium-containing materials are widely used in the ceramics and glass industry; in the production of aluminium, pharmaceuticals, batteries and certain weapons, and in its elemental form, as a nuclear reactor coolant [2]. Lithium occurs in numerous minerals and is mobilised by weathering processes, transported into soils, from which it is taken up by plants and enters easily into the food chain. In animals, lithium is not bioaccumulative and their compounds generally have a low toxicity [1]. Although lithium has thus far not been shown to serve as a required cofactor of any enzyme or enzymatic transport system, it is increasingly regarded as an essential trace element for animals and humans [3, 4]. At pharmacological dosages, lithium exerts interesting in vivo effects, causing it be used in the therapy of manic–depressive psychosis [5]. As lithium appears to alter neurotransmission at a synaptic level in the brain, carbonate and other lithium salts have been used in psychiatry for over 50 years mainly to moderate mood swings, although its mechanism of action is still poorly understood. At extremely low levels of intake, symptoms of lithium deficiency were evident in experimental animals, affecting the endocrine, cardiovascular, neuromuscular, renal and dermatological systems [1, 4].

With a few exceptions [5–8], the increasing use of lithium in recent years has not provoked many studies dealing with the toxic influence of this metal to higher plants and their tolerance to its presence. Lithium is taken up by all plant species, and although it appears not to be crucial for proper growth and development, stimulation of plant growth has been observed [1]. Furthermore, there is some evidence that lithium can have some metabolic functions in halophytes, but at high concentration levels in the soil, lithium is toxic to all plants, triggering chlorosis-like symptoms. Lithium shares the potassium (K^+^) transport carrier; therefore, it is easily transported to the above-ground parts of the plants and accumulates mainly in leaf tissues [4]. Various plants present different absorbing abilities and tolerance levels to its high concentration in soil solution. Lithium-accumulating plants belong to the *Asteraceae* and *Solanaceae* families, as well as to the group of halophilic plants [3, 4]. Citrus plants are relatively sensitive to lithium [1]. An excess of lithium can inhibit the rhythmic movements of plants, disrupts normal pollen development and blocks pollen germination [5]. Moreover, lithium ions can modulate growth and gravitropic response of maize roots [9] and affect cold-induced dephosphorylation of microtubules in spinach cells [10]. The most widely accepted model for lithium action is the ‘inositol depletion hypothesis’ [11], which is based on the inhibition of inositol monophosphatases by Li^+^ ions, leading to a depletion of cellular inositol levels and inhibition of the inositol cycle and calcium signalling. Plant inositol monophosphatases are Li^+^-sensitive, and the influence of lithium on plants has also been interpreted in terms of an inhibition in the inositol cycle and calcium-dependent signalling pathways [5].

The aim of this study was to learn more about the biological activity and accumulation of lithium in monocot (maize) and dicot (sunflower) plants. In this study, the influence of different concentrations of lithium in the nutrient solution on the plant biomass, contents of assimilation pigments, leaf area, levels of lipid peroxidation as well as lithium and potassium contents in shoots were examined.

## Materials and Methods

### Plant Material and Growth Conditions

Seeds of sugar maize (*Zea mays* L. var. *saccharata* Kcke) cv. Zlota Karlowa and common sunflower (*Helianthus annuus* L.), purchased from the Lublin Seed Centre, were germinated in germination rolls, between two layers of moistened filter paper, for 7 days at 25°C. After germination, the healthiest, best-developed seedlings were transferred to 1-dm^3^ glass jars (two per jar) filled with 1.5 times strength Hoagland’s II nutrient solution supplemented with the essential microelements [12]. The nutrient solution pH was adjusted to 6.0 using diluted NaOH. Thereafter, lithium in the form of LiCl was added to the medium at the following concentrations (in milligrams of Li per cubic decimetre): 0 (control), 5, 25 or 50. Plant growth proceeded in a climate-controlled chamber (Sanyo, model MRL 350HT) under photosynthetic photo flux density of 270 μmol m^−2^ s^−1^, 14-h day length, temperature of 25/20°C (day/night) and with a relative humidity of 70–75 %. After 14 days of growth under different lithium concentrations, the leaf areas of plants, photosynthetic pigment contents and levels of lipid peroxidation were examined. Then, the control and Li-treated plants were harvested and separated into roots and shoots, and then the fresh weights (FW) were determined immediately after harvest. Samples of the above-ground parts of the harvested plants were dried to a constant dry weight (DW) at 105°C, and then they were ground in preparation for lithium and potassium analysis.

### Determination of Leaf Areas and Photosynthetic Pigment Contents

Fresh second leaves were scanned using CI-202 laser area metre (CID Bio-Science, USA) and the leaf area was expressed in square centimetres (cm^2^). The chlorophylls *a* and *b* together with all carotenoids (xanthophylls + carotenes) were determined spectrophotometrically following the procedures of Lichtenthaler and Wellburn [13]. The samples were collected from second true leaves and the pigments were extracted from fresh leaf discs via homogenization in 80 % (*v*/*v*) acetone. Absorbance of the resulting extracts was measured at 646, 663 and 470 nm.

### Lipid Peroxidation Assay

Lipid peroxidation has been acknowledged as a major cause of cellular injury in many biological systems of plant and animal origin. The lipid peroxidation of membranes was estimated by measuring the malondialdehyde (MDA) content, which is a by-product of lipid peroxidation in tissue extracts. MDA contents were assayed following the method of Heath and Packer [14] with minor modifications. In order to determine the MDA content in roots and leaves, tissues (500 mg) were ground in 4.5 cm^3^ of 0.1 % (*w*/*v*) trichloroacetic acid (TCA) and centrifuged at 10,000 rpm for 10 min. Then, 4 cm^3^ of 20 % TCA containing 0.5 % of thiobarbituric acid (TBA) (*w*/*v*) was added to 1 cm^3^ of the obtained supernatant. The solution of TCA + TBA was enriched using butylated hydroxytoulene to prevent MDA formation during the assay, which could result in falsely elevated TBA reactivity. The mixture was heated to 95°C for 30 min, quickly cooled on ice and re-centrifuged for 10 min. The absorbance of solution was measured at 532 and 600 nm wavelength against blank. The amount of MDA–TBA complex (red pigment) was calculated from the extinction coefficient 155 mM^−1^ cm^−1^ and expressed as nanomole of MDA per gram FW.

### Lithium and Potassium Content Determinations

The lithium content in the plant’s shoots was determined by the inductively coupled plasma optical emission spectrometry (ICP-OES) method and the potassium content was determined applying the AAS technique.

### Statistical Analysis

The experimental design was randomised with four treatments and four replications per each treatment. The experiment was repeated three times under the same conditions. A one-way ANOVA test was used to compare the obtained results followed by a post hoc multiple comparisons of means using Tukey’s test. All statistical calculations were performed using StatSoft Statistica version 6.0, and differences at *p* < 0.05 were considered significant.

## Results

### Growth Parameters and Photosynthetic Pigment Accumulation

**Fig. 1:**
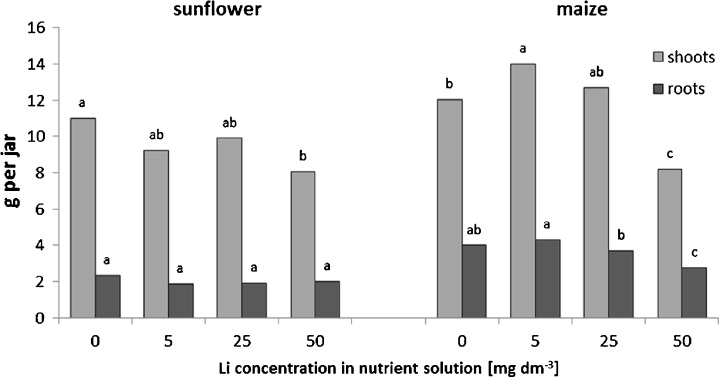
Effects of increasing lithium concentrations in nutrient solutions on the fresh biomass in sunflower and maize. Mean values for each organ and plant species, marked with the *same letters*, are not significantly different at *p* < 0.05

At the highest applied lithium doses (50 mg Li dm^−3^ of nutrient solution), a significant reduction of the shoot biomass occurred in both examined species, by 27 and 32 % in sunflower and maize, respectively. In maize, in the presence of 5 mg Li dm^−3^, an increase (15 %) in biomass accumulation was noted, as compared to the control plants. There was no significant effect of lithium treatment at a concentration range of 5–50 mg Li dm^−3^ on the fresh weight of roots, except of significant decrease (31 %) of maize root biomass in the presence of 50 mg Li dm^−3^ (Fig. 1).

**Fig. 2:**
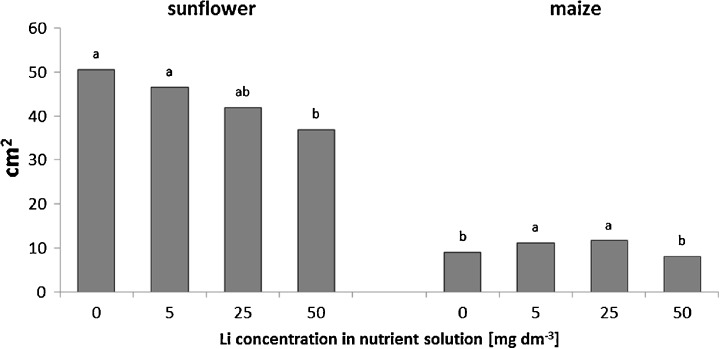
Effects of increasing concentrations of lithium in nutrient solution on the leaf areas of sunflower and maize. Mean values for each species, marked with the *same letters*, are not significantly different at *p* < 0.05

**Fig. 3:**
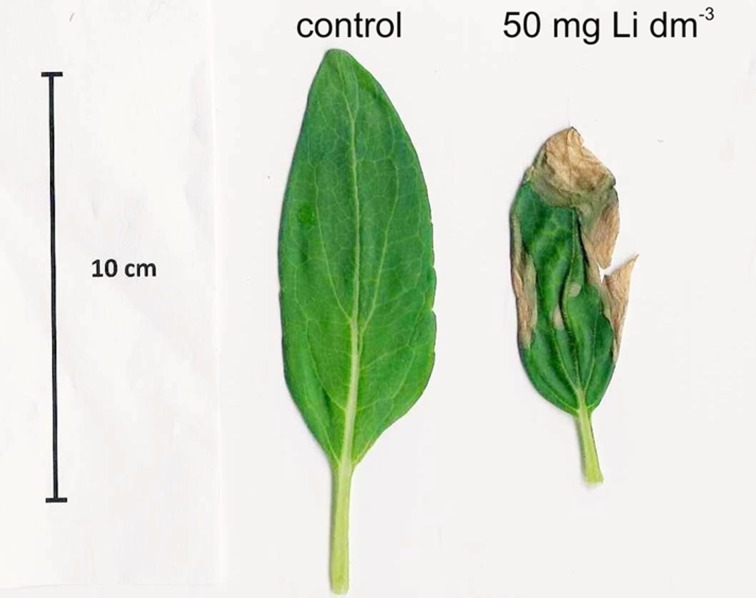
Necrotic spots on the oldest leaf of a sunflower exposed to 50 mg Li dm^−3^ for 12 days

As a result of the lithium treatments, the assimilating organs area of sunflower plants decreased with increasing concentrations of lithium. However, a significant reduction (27 % compared to control plants) was found only at the highest lithium dose examined (50 mg Li dm^−3^) (Fig. 2). Furthermore, sunflower plants supplied with the highest lithium concentrations initially induced the formation of necrotic spots of various sizes in older leaves (Fig. 3), which resulted in almost complete necrosis of these leaves after 14 days of exposure to 50 mg Li dm^−3^. In maize, the supplementation of the nutrient solution with 5 and 25 mg Li dm^−3^ caused the leaf area to increase by 22 and 29 %, respectively (Fig. 2).

**Fig. 4:**
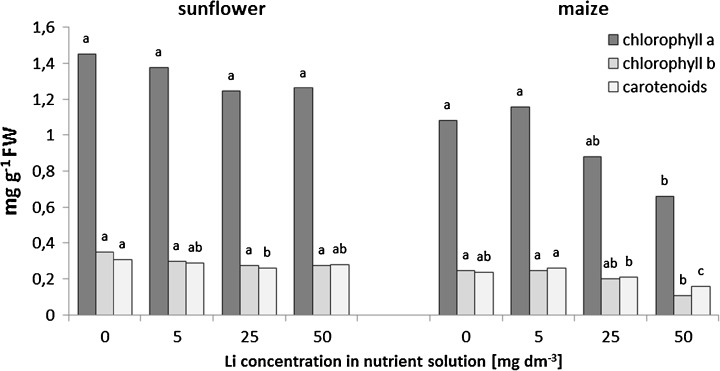
Effects of increasing lithium concentrations in the nutrient solutions on the pigment content in sunflower and maize. Mean values for each pigment class and plant species, marked with the *same letters*, are not significantly different at *p* < 0.05

The obtained results indicate that the supplementation of the nutrient solution with 5–50 mg Li dm^−3^ does not significantly affect the chlorophyll *a* and *b* concentrations in the sunflower leaves (Fig. 4). However, the chlorophyll *a*/*b* ratio increased from 4.3 in the control to 4.7–4.8 in Li-affected plants (data not shown). In spite of that, the carotenoids content significantly decreased by 16 % after the enrichment of a nutrient solution with 25 mg Li dm^−3^, if compared to the sunflower plant control. In maize, however, a significant decrease in chlorophylls *a* and *b* content by 47 and 43 %, respectively, in comparison with the control plants, under highest used lithium concentrations (50 mg Li dm^−3^), was noted (Fig. 4). Furthermore, a decrease in the chlorophyll *a*/*b* ratio from 4.4 in the control to 4.0 in 50 mg dm^−3^ Li-treated plants was observed (data not shown). Additionally, the carotenoid level in maize leaves also decreased by 50 mg dm^−3^ lithium supplementation and reached 67 % of the control value (Fig. 4).

### Levels of Lipid Peroxidation

**Fig. 5:**
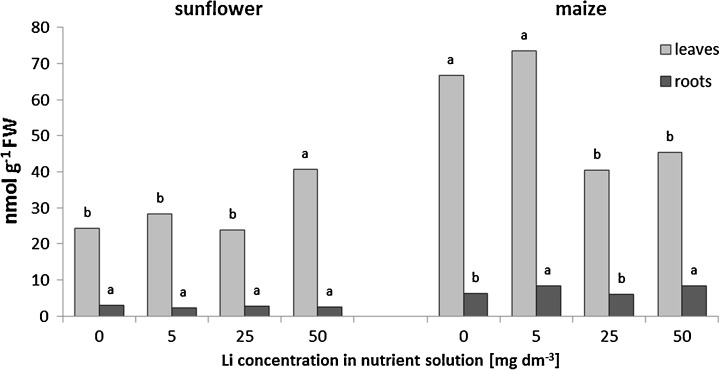
Effects of increasing lithium concentrations in the nutrient solutions on the MDA contents in sunflower and maize. Mean values for each organ and plant species, marked with the *same letters*, are not significantly different at *p* < 0.05

The levels of lipid peroxidation of the cell membranes of the sunflower leaves increased significantly by 68 % in the presence of 50 mg Li dm^−3^, suggesting that disturbances of membranes integrity occurred. Nonetheless, the exposure to lithium caused no significant changes of the MDA contents in the sunflower roots. The value of this parameter decreased by 39 and 32 % in maize leaves after an application of 25 and 50 mg Li dm^−3^ to the medium, respectively. On the other hand, in the roots of maize, there was a significant increase (32 %) in the MDA level in response to 5 and 50 mg Li dm^−3^ (Fig. 5).

### Lithium and Potassium Contents of Shoots

The results of the Li^+^ and K^+^ analysis in shoots are presented in Table 1. The lithium content, which was low in the control plants, greatly increased after LiCl treatment in both plant species. Increasing lithium concentrations in the growth medium resulted in a progressive dose-dependent increase in lithium content in shoots. Sunflower plants accumulated considerably greater amounts of this metal than maize. The lithium content in sunflower and maize shoots reached 3,292 and 695 mg Li kg^−1^ DW, respectively, after supplementation of the nutrient solutions with 50 mg Li dm^−3^.

**Table 1:** Effects of increasing lithium concentrations in nutrient solutions on the lithium and potassium contents in shoots of sunflower and maize

Plant species	Li concentration in nutrient solution (mg dm^−3^)	Content of elements (mg kg^−1^DW)	
		Li^+^	K^+^
Sunflower	0	0.9 ± 0.049 a	9.82 ± 0.0778 a
	5	422.5 ± 4.949 b	9.88 ± 0.0990 a
	25	432.0 ± 11.313 b	9.66 ± 0.0495 a
	50	3,292.0 ± 24.051 c	10.90 ± 0.0636 b
Maize	0	4.11 ± 0.007 a	9.59 ± 0.0354 a
	5	72.90 ± 0.566 b	9.71 ± 0.0424 a
	25	438.00 ± 14.142 c	9.16 ± 0.0990 a
	50	695.00 ± 12.828 d	8.55 ± 0.0635 a

Mean values for each element and plant species marked with the same letters are not significantly different at *p* < 0.05

Both plant species accumulated similar amounts of potassium in their shoots at increasing lithium levels, except at the highest level of Li (50 mg dm^−3^), at which sunflower plants accumulated more potassium than maize. Moreover, in sunflower plants, the potassium contents were significantly higher than in the control plants after exposure to the nutrient solution with 50 mg Li dm^−3^.

## Discussion

Our results demonstrate that growing sunflower and maize plants in the presence of 50 mg Li dm^−3^ causes a marked decrease in the fresh biomass of both plants, suggesting that this lithium concentration is toxic to both plant species. Furthermore, in maize under the highest used lithium concentration (50 mg Li dm^−3^), a significant decrease in the content of chlorophylls *a* and *b* and of carotenoids was noted, whereas in sunflower plants, the content of photosynthetic pigments was not affected by the presence of lithium in the nutrient solution. However, the treatment of sunflower plants with 50 mg Li dm^−3^ provoked a reduction of the leaf area and progressively induced the formation of necrotic spots of various sizes on older leaves. Naranjo et al. [5] observed the formation of necrotic lesions and leaf curling in tobacco plants exposed to 50 mM LiCl. These alterations were more obvious also in older leaves and resulted from preferential Li^+^ accumulation in those leaves. Kent [15] suggests that lithium stored in the oldest leaves is immobile and cannot be translocated to other organs. Moreover, Zeller and Fuller [16] have found that lithium ions are almost immobile in the phloem tissues of wheat plants. Necrotic spots are typical for incompatible (avirulent) plant–pathogen interactions, and the induction of necrotic spots in Li-treated plants seems to be mediated by ethylene [5].

In maize, a growth-stimulating effect of lithium was noted at a concentration of 5 mg Li dm^−3^. McStay et al. [6] stated that at 4 ppm Li, plant height, first trifoliate fresh weight and leaf area of *Phaseolus vulgaris* L. increased significantly. However, at concentrations greater than 4 ppm, stomatal diffusive resistance increased, indicating partial stomatal closure, and provoked disturbances in water relations of plants. Li et al. [8] showed that up to 30 mM LiCl exposure, lithium has almost no effect on the germination and growth parameters of *Brassica carrinata* seedlings. However, above this concentration, toxicity rapidly increased along with the reduction of germination rate, fresh weight, root length, as well as seeing a significant decrease in the chlorophyll content. Lithium exposure also affected the lipid and phenolic composition of *B*. *carrinata* seedlings.

Data from ICP-OES showed that the lithium content in shoots directly and meaningfully correlated with concentrations of this element in the nutrient solution; however, sunflower plants accumulated considerably greater amounts of this metal (about five to sixfold) than maize. Surprisingly, in plants grown in the presence of 25 mg Li dm^−3^, the lithium content was similar in both species. The lithium level is considered to be higher in dicotyledonous plants than in monocotyledonous ones [17]. Lithium in plants and animals interacts with sodium and potassium and with enzymes requiring magnesium [1]. Therefore, in the present study, we determined the potassium content in shoots of plants. However, no significant changes were found between the control and Li-treated plants, except for an increase in the K^+^ level in sunflower plants grown in the presence of 50 mg Li dm^−3^. Naranjo et al. [5] reported that LiCl treatments caused a rapid depletion of K^+^ from the tobacco leaves, but this decrease of the K^+^ level did not correlate with the intensity of visual Li-induced toxicity symptoms. Despite that, Magalhães et al. [18] did not state any significant changes in the K^+^ level in lettuce and watercress exposed to lithium at a concentration range of 0.1–2 mM but observed a decrease in the K^+^ content in radish under the same conditions.

To our knowledge, the effect of the applied lithium salts on lipid peroxidation in plant tissues has not been studied earlier; although in animal tissues, it is widely determined as a marker of oxidative stress under lithium supplementation [e.g. 19–21]. In our research, the level of lipid peroxidation (as evidenced by MDA levels) of the cell membranes in leaves and roots of sunflower and maize plants, respectively, increased significantly in the presence of 50 mg Li dm^−3^ which suggests the disturbances of membrane integrity. Elevated lipid peroxidation generally positively correlated with a decline in the plant’s biomass. It has been suggested that oxidative stress, resulting from an excessive formation of reactive oxygen species, is one of the important mechanisms of the toxic effects of lithium ions in animals [19]. Thus, we suppose that the excess of lithium can exert a similar effect in plants. However, the MDA content in maize leaves was found to be significantly reduced after an application of 25 and 50 mg Li dm^−3^ when compared to control plants. Therefore, changes in the MDA content seem to be not fully unambiguous and are organ-dependent and species-specific.
